# Interactions between *Aspergillus fumigatus* and Pulmonary Bacteria: Current State of the Field, New Data, and Future Perspective

**DOI:** 10.3390/jof5020048

**Published:** 2019-06-12

**Authors:** Benoit Briard, Gaëtan L. A. Mislin, Jean-Paul Latgé, Anne Beauvais

**Affiliations:** 1 Aspergillus Unit, Institut Pasteur, 75015 Paris, France; benoit.briard@stjude.org (B.B.); jean-paul.latge@pasteur.fr (J.-P.L.); 2 UMR 7242 Biotechnologie et Signalisation Cellulaire, CNRS-Université de Strasbourg, 67400 Illkirch-Graffenstaden, France; gaetan.mislin@unistra.fr

**Keywords:** interaction, *Aspergillus*, microbiota, cystic fibrosis, *Pseudomonas*, cell wall, phenazine, rhamnolipid, pyochelin, volatile

## Abstract

*Aspergillus fumigatus* and *Pseudomonas aeruginosa* are central fungal and bacterial members of the pulmonary microbiota. The interactions between *A. fumigatus* and *P. aeruginosa* have only just begun to be explored. A balance between inhibitory and stimulatory effects on fungal growth was observed in mixed *A. fumigatus–P. aeruginosa* cultures. Negative interactions have been seen for homoserine-lactones, pyoverdine and pyochelin resulting from iron starvation and intracellular inhibitory reactive oxidant production. In contrast, several types of positive interactions were recognized. Dirhamnolipids resulted in the production of a thick fungal cell wall, allowing the fungus to resist stress. Phenazines and pyochelin favor iron uptake for the fungus. *A. fumigatus* is able to use bacterial volatiles to promote its growth. The immune response is also differentially regulated by co-infections.

## 1. Introduction

*A. fumigatus* is the most important opportunistic aerial fungal pathogen. It is a ubiquitous microorganism in the ambient air which is responsible for pulmonary infections resulting from the inhalation of conidia [1]. Several types of aspergillosis can be seen, depending on the immune status or the underlying disease or the environment of the patient [2,3,4,5,6,7].

Most of studies performed to date to understand the physiopathology of aspergillosis have been focused on *A. fumigatus* alone. In nature, *A. fumigatus* is never alone, but is always present in microbial communities [8,9]. Are the lung microbiota partners of *A. fumigatus* influencing the pathogenic life of this fungal species? In this review, the analysis of the relationship between *Aspergillus* and *Pseudomonas aeruginosa* (and a few other microbiota bacteria) resulting either from direct contact between the two microorganisms or through secreted bacterial compounds are presented. In addition, unpublished data on the role of pyochelin of *P. aeruginosa* siderophore on *A. fumigatus*, as well as the immunological consequences of co-infections, have been added to draw up a more comprehensive picture of these interactions. 

## 2. The Lung Microbiota in Different Clinical Situations

The respiratory tract is the human body site with the largest surface harboring interacting bacteria and fungi. DNA-based culture-independent techniques are now used to identify the microbial species harbored in the lung epithelial surface. Lung microbiota has been determined mainly in cystic fibrosis (CF) and chronic obstructive pulmonary disease (COPD) patients, where it is easier and safer than in other patients to collect human specimens. Among these patients, the fungal mycobiome composition has been analyzed in bronchoalveolar lavages or sputum samples [6,7,8,10,11,12]. Despite discordance in the results, mainly due to a lack of standardization of the methods, many fungal species were isolated from the respiratory tract, including *Candida albicans*, *A. fumigatus*, *A. flavus*, *Geotrichum* sp., *Pneumocystis jirovecii*, *Malassezia sp.*, *Scedosporium apiospermum* and *Exophiala dermatitidis*. Many independent studies have identified *A. fumigatus* as the principal colonizing fungus in COPD or CF patients [3,9,12,13]. In these patients, the altered structure of the lung epithelium and the changes in the composition of the normal flora in the respiratory airway can promote *A. fumigatus* adhesion and persistence [3]. Detection and quantification of microbial species have also revealed that the lung comprises a large number of bacterial taxa. In CF, the most abundant bacteria found in sputum samples from patients with end-stage disease are usually from a small group of known CF pathogens including *Pseudomonas aeruginosa*, *Stenotrophomonas maltophilia*, *Staphylococcus aureus*, *Burkholderia cenocepacia* and non-tuberculous *Mycobacteria abscessus* [14,15]. *S. aureus* commonly infects pediatric CF patients, while *P. aeruginosa* dominates the bacterial community in adult patients [16]. Eradication therapy for *P. aeruginosa* increased the rate of infection with *S. maltophilia* [16]. In COPD, concomitant isolation of *A. fumigatus* and *P. aeruginosa* was also reported [17]. The composition of the COPD lung microbiota identified as the main phyla *Actinobacteria*, *Firmicutes* including *Streptococcus* sp and *Proteobacteria* including *P. aeruginosa* [8]. Even though the role of the virome was not specifically investigated in relation to *A. fumigatus*, several reports have indicated that viruses may have an impact (although probably indirect) on *Aspergillus* infections. Cytomegalovirus and Epstein-Barr virus have been known for years to be associated with *Aspergillus* infection. More recently, the influenza virus was recognized as a strong inducer of aspergillosis [18]. Moreover, sputa collected from CF patients contained high number of phages which can be stimulated by biofilm or hypoxia conditions during bacterial growth [19]. Many *A. fumigatus* strains are strains host viruses intracellularly suggesting a putative role in horizontal gene transfer [20].

Associations between *A. fumigatus* and lung infecting bacteria specifically linked with COPD have not been studied until now. In contrast, the joint presence of *A. fumigatus* and *P. aeruginosa* has been investigated in CF patients, particularly in the chronically infected oldest patients [21]. *P. aeruginosa* evolves in CF airways, producing variants, such as those resulting in mucoid colony types which are adapted to chronic residence there. In most patients, *A. fumigatus* colonization is preceded by *P. aeruginosa* infection [22]. It has been shown that more rapid decline in pulmonary function due to an increase of the inflammatory response, and more severe clinical outcomes have been observed in CF patients simultaneously infected with *A. fumigatus* and *P. aeruginosa*, when compared to *P. aeruginosa* infection alone [23]. However, in a murine pulmonary model, immunosuppressed mice co-infected with *A. fumigatus* and *P. aeruginosa* had a higher survival rate than mice infected by *A. fumigatus* alone, and the score for hyphal growth in the *A. fumigatus* infected mice after inoculation of *P. aeruginosa* was significantly lower than that of mice infected by *A. fumigatus* alone, suggesting that *P. aeruginosa* may secrete antifungal compounds or stimulate the immunological antifungal host response [24]. The transition of *P. aeruginosa* from non-mucoid to mucoid, a process that occurs with time in CF disease, increased its virulence inside the patient body but decreases *P. aeruginosa* inhibitory effect on *A. fumigatus* explaining why establishment of *A. fumigatus* in the airways occurs later in CF disease [25]. Bacteria other than *P. aeruginosa* have been noticed to accompany *A. fumigatus* during lung infection. Significant associations have been reported between the upper airways colonization by *A. fumigatus* and the presence of *S. maltophila* [26]. *S. maltophila* is an important hospital-associated pathogen; it is not highly virulent, but its environmental dissemination and resistance to selective pressure antibiotics promote its opportunistic pathogenicity in immunocompetent patients or CF patients. However, the clinical impact of this association has not been evaluated yet. No studies were reported on interactions between *A. fumigatus* and *S. aureus* or *B. cenocepacia* in CF context. The reasons are that CF patients are infected by *A. fumigatus* later after *S. aureus* infection, and negative associations were reported between *A. fumigatus* and *B. cenocepacia*, which means that the two bacterial species were never found at the same time as *A. fumigatus* in CF patients [16,27]. However, outside of the lungs, co-colonization of *A. fumigatus* and *S. aureus* on contact lenses can cause co-infectious keratitis [28]. The presence of *A. fumigatus* has been mentioned to be associated with an increased risk of non-tuberculous *Mycobacteria* infection, especially *M. abscessus*, which is one of the most clinically virulent and antibiotic-resistant bacterial species [15].

## 3. Interactions In Vitro between Lung Bacteria and *A. fumigatus*

### 3.1. Methodologies for Studying Bacterial–Fungal Interactions In Vitro

Due to the difficulty of studying bacteria–*A. fumigatus* interactions in a host, interactions were first investigated in in vitro models. Of course, these in vitro models cannot mimic in vivo infections, because it is still unknown how the bacteria and *A. fumigatus* enter in contact within the host. The relevance to host airway of these in vitro models is that they are the best way to study the molecules produced by one partner to control the growth of the other. Some of these molecules are secreted, acting at distance, and can be specifically produced in response to the presence of the partner. These models can help the understanding of what happens in vivo during co-infections. However, lack of standardization in the methods induced high variability in the data from one study to another one [29]. In vitro models of interaction are highly dependent on the bacterial and fungal inoculum, the strains used, the composition of the medium, solid or liquid medium, shaken conditions including a membrane separating the two microorganisms or unshaken conditions, temperature and pH [29]. To analyze in the most efficient way the interactions between bacteria and *A. fumigatus*, it is necessary to avoid the overgrowth of one partner over the other since it may be responsible for an excessive killing of the second partner. The best experimental settings to study direct interactions during mixed *P. aeruginosa*–*A. fumigatus* biofilms were obtained at 37 °C on Sabouraud dextrose broth with 10^6^ conidia/mL inoculated 18 h before the inoculation of 10^6^ *P. aeruginosa*/mL and further incubation for 24 h [30], or on RPMI-MOPS agar plates inoculated with 2 × 10^7^ *A. fumigatus* conidia and at the same time 5 μL of 2.5 × 10^5^ *P. aeruginosa* spotted in the center of the Petri dish [31]. In this last model, the mixed biofilm was observed at the junction between the bacterial and the fungal colonies. The best mixed *S. maltophila*–*A. fumigatus* biofilm was obtained on RPMI-MOPS supplemented with 10% bovine fetal serum with simultaneous inoculation of 10^5^ *A. fumigatus* conidia/mL and 10^6^ *S. maltophila*/mL and incubation for 24 h [26]. The best mixed *S. aureus–A. fumigatus* biofilm was obtained on RPMI supplemented with 2% glucose inoculated with 10^5^ *A. fumigatus* conidia/mL and 10^8^ *S. aureus*/mL and incubated for 4 h. The supernatant in this last biofilm model was eliminated to remove non-adherent cells and replaced by fresh RPMI. The incubation was pursued for 24 h [28].

### 3.2. Cell–Cell Interactions

#### 3.2.1. Fungal Adaptation to the Presence of Bacteria

• Production of a protective extracellular matrix

In the mixed in vitro bacteria–*A. fumigatus* models presented above, bacterial cells were always seen adhering to the fungal hyphae [26,31]. Galactosaminogalactan (GAG), which is a virulent factor of *A. fumigatus* synthesized on the surface of the cell wall and in the extracellular matrix [32], was responsible for the binding of *P. aeruginosa* to the hyphae and in the biofilm. The production of this polysaccharide increased in response to the bacterial assault against the fungus [31]. Moreover, in *P. aeruginosa*–*A. fumigatus* mixed biofilm, an electron-dense material was observed on the extracellular matrix of the hyphae, and was found to be dihydroxynaphthalene (DHN)- and pyo-melanin [31]. DHN-melanin protects *A. fumigatus* conidia against a range of aggressions such as desiccation, UV light or oxidant agents. During infection, DHN-melanin inhibits the non-canonical autophagy pathway termed LC3-associated phagocytosis (LAP) that promotes phagolysosomal fusion and fungal killing [33]. Pyomelanin is synthesized via the tyrosine degradation pathway. Cell wall stress induced the production of pyomelanin [34]. The presence of these three molecules in the extracellular matrix should protect *A. fumigatus* against host response. The appearance of these phenotype in *A. fumigatus* requires a tight contact with the bacteria in the host airway, which is likely, since both microorganisms are recovered in sputum samples [6,7].

• Production of a thick cell wall/role of bacterial dirhamnolipids and maltophilin

One morphogenetic modification of the presence of *P. aeruginosa* or *S. maltophila* is a thickening of the cell wall in response to the bacterial stress [26,31]. In addition, in the presence of bacteria, the hyphae are highly ramified, with short ramifications at the tips. The molecules secreted by *P. aeruginosa* or *S. maltophila* responsible for increasing the thickness of the fungal cell wall are dirhamnolipids and maltophilin, respectively [31,35,36,37]. 

**Figure 1:**
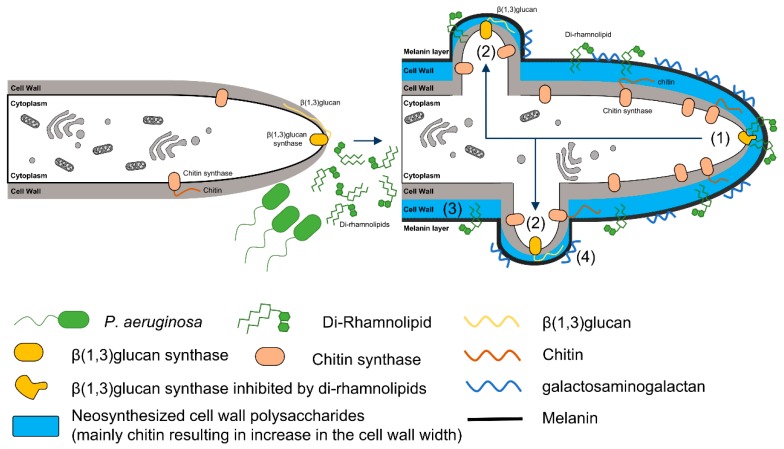
Diagram illustrating the mode of action of *P. aeruginosa* dirhamnolipids on *A. fumigatus* growth. Dirhamnolipids inhibit β1,3 glucan synthase (GS) at the hyphal tip (1). This inhibition stimulates the formation of new apices (2), containing active GS which will be further inhibited by the dirhamnolipids, giving the multibranched phenotype with short apical cells. The inhibition of the β1,3 glucan synthesis is compensated by an increase in chitin synthesis (3). Dirhamnolipids also induce melanin and galactosaminogalactan production in the extracellular matrix (4).

A mixture of mono and dirhamnolipids are produced by *P. aeruginosa* and other *Pseudomonas* sp. They are non-diffusible molecules overproduced during stress conditions and are components of the biofilm. For a long time, dirhamnolipids were only known to display tensioactive properties, conferring to the molecules anti-microbial and anti-human cell activity [37,38,39,40,41]. The biosurfactant activity of the dirhamnolipids induces the disorganization of the plasma membrane, resulting in quantitative changes in phospholipid headgroup in *Bacillus subtilis* [40]. In addition, dirhamnolipids are responsible for the induction of galactosaminogalactan and melanin production, hyphal wall thickness and ramifications by *A. fumigatus* [31]. Dirhamnolipids induced the production of an increased concentration of cell wall chitin (3-fold increase) in compensation for β1,3 glucan synthase inhibition. The branched lipid tail with β-hydroxy-fatty acids of the dirhamnolipids is essential for its activity on the β1,3 glucan synthase activity. The effect of dirhamnolipids is never fungicidal and is very reminiscent of the echinocandin effect. Echinocandins are clinical antifungal lipopeptides used in the treatment of invasive pulmonary aspergillosis which target the β1,3 glucan synthase [42]. Echinocandins are also fungistatic against *A. fumigatus* and the hyphae are also highly ramified with short ramifications at the tips [42]. However, we demonstrated that the di-rhamnolipid site of action on the β1,3 glucan synthase is different from the one of echinocandins. The mode of action of dirhamnolipids on *A. fumigatus* is summarized in Figure 1.

Maltophilin, which is produced by *S. maltophila*, has been also shown to increase *A. nidulans* cell wall thickening and chitin levels, in response to defective sphingolipid metabolism [36]. Maltophilin induces the accumulation of sphingolipid intermediates which may stimulate cell wall synthesis by activating cell wall integrity pathways. 

#### 3.2.2. Extracellular Soluble Molecules

Various studies have confirmed the presence of bacterial quorum-sensing molecules in the sputum of CF patients [43,44,45]. These molecules have low molecular weight and different structure, and are known to modulate the pathogenicity of pathogens [46]. The best-known and most-studied quorum-sensing molecules are from *Pseudomonas* spp. These include homoserine lactones (HSLs), quinolones (PQS) and phenazines [47,48,49]. The two siderophores pyoverdine and pyochelin are also known virulence factors in *P. aeruginosa* [50,51]. The role of these compounds in the communication between *P. aeruginosa* and *A. fumigatus* has been investigated.

• Homoserine Lactones and Quinolones

HSLs are known *P. aeruginosa* virulence factors as they induce the production of IFNγ and the disruption of NFκB, resulting in an inflammatory process [52]. HSLs significantly restricted the capacity of *A. fumigatus* to form hyphae and reduced the biomass of the fungal biofilm [47], but their mode of action is not yet understood. The effect of HSL is similar on *C. albicans*, as it inhibits hyphal growth and consequently the biofilm formation [53]. PQS also altered biofilm biomass and structure by reducing the attachment of the conidia to the polystyrene plates and blocking the germination [48]. In comparison, *C. albicans* quorum-sensing molecule farnesol inhibits *P. aeruginosa* PQS biosynthesis [54], but to date, nothing is known about a similar *A. fumigatus* quorum-sensing molecule acting on bacterial production of HSLs or PQS [55].

• Phenazines

Four major phenazines have been described: pyocyanin (PYO), phenazines-carboxamide (PCN), phenazines-carboxylic acid (PCA) and 1-hydroxy-phenazine (1HP) [56]. They are heterocyclic redox-active compounds. They are small diffusible quorum-sensing molecules that easily penetrate all kinds of cells, including *A. fumigatus* conidia, as soon as they undergo swelling and germination [49]. They are considered one of the strongest virulence factors of *P. aeruginosa* against a broad range of target organisms and host immune cells. Their antagonistic effects are attributed to their redox potential [49,57,58,59]. Reduced phenazines are oxidized in the fungal cell by oxygen and NADPH through a NapA-dependent oxidative stress response, generating ROS [57]. The main ROS target of phenazines is the mitochondria in the hyphal cells [49]. In *A. fumigatus* cells, all phenazines at high concentrations induce the production of ROS and reactive nitrogen species (RNS) by mitochondria, which are released into the cytoplasm, leading to the fungal death. Sod2, which is the mitochondrial superoxide dismutase in *A. fumigatus*, is essential for ROS and RNS resistance induced by phenazines [49]. *A. fumigatus* has been shown to metabolize phenazines [60]. PYO is bio-transformed into phenazine dimers and PCA into 1-HP which is then metabolized into 1-methoxyphenazine (1-MP) and phenazine-1-sulfate. 1-MP also has an antifungal activity, while phenazine-1-sulfate does not. The capability of fungi to metabolize a variety of molecules is a known mechanism to detoxify and mineralize compounds, particularly in fungal–bacterial interactions. However, 1-MP was a more potent inhibitor than PCA, suggesting that 1-MP was an intermediate in the detoxification process [60].

1-HP was found to be the most active phenazine against *A. fumigatus*. In addition to ROS and RNS production, its high inhibitory activity is due to a specific iron chelation property [49]. Indeed, 1-HP-iron complex causes *A. fumigatus* iron starvation and consequently induces the production of fungal extracellular siderophores fusarine C (FsC) and triacetylfusarinine C (TAFC). Moreover, all genes required for adaptation to iron starvation (*HAPX*, *SIDA*, *SIDF*, *SIDG* and *MIRB*) are induced by 1-HP, whereas genes encoding iron-dependent proteins are repressed (*ACOA* and *CYCA*) [49,60]. 

Interestingly, phenazines have a dual effect on *A. fumigatus* [49]. In addition to the antifungal activity at high concentrations, an enhancing fungal activity has been described for PYO, PCN and PCA at low concentrations and in iron-starved environment. This effect is due to the ability of these phenazines to reduce ferric iron Fe^3+^ to ferrous iron Fe^2+^, which is taken up by the bacterial cells via the ferrous iron transporter. This characteristic is also an advantage for *A. fumigatus* growth. Fe^2+^ is more soluble than Fe^3+^ and can penetrate the fungal cell by low affinity ferrous iron uptake pathway, involving the FetCp/FrA permease complex [49]. Similarly, it has been reported that phenazines were responsible for enhanced *A. fumigatus* conidiation at sub-inhibitory concentrations, which is an operative stress response pathway [57].

By comparison, in *C. albicans*, only the antifungal activity of phenazines via the production of ROS altering respiratory activity has been reported so far [59]. In this way, *P. aeruginosa* causes *C. albicans* to secrete more fermentation products that are readily used by the bacteria to enhance its own growth and survival.

• Pyoverdine and Pyochelin Siderophores

An important factor allowing host colonization is the efficient uptake of iron by the bacterium. In the mammalian host, iron is not freely available, since it is either present in the heme molecule found in hemoproteins or strongly chelated by the extracellular proteins transferrin and lactoferrin. *P. aeruginosa* secretes two siderophores to acquire iron, pyoverdine, the high affinity siderophore (pFe = 27), and pyochelin, the low affinity siderophore (pFe = 16) [51]. Both are also chelators of other divalent metals, such as zinc (Zn^2+^) and copper (Cu^2+^), which are cofactors of enzymes with crucial roles in bacterial metabolism [61]. It has been demonstrated that *P. aeruginosa* first produces pyochelin and switches to pyoverdine production when concentration of iron becomes really low [62]. The role of pyoverdine and pyochelin in a murine model of *P. aeruginosa* and *C. albicans* gut co-colonization and the antifungal activity of pyoverdine on *A. fumigatus* were recently analyzed [50,63]. No studies have reported the role of pyochelin on *A. fumigatus*. Since we had unpublished data on the effect of pyochelin on *A. fumigatus,* we decided to include these data for a more comprehensive understanding of the role of the *P. aeruginosa* siderophores on *A. fumigatus*. For this purpose, the following paragraph on pyochelin is in a different style than the rest of the review, since it presents in a more detailed way the role of pyochelin on *A. fumigatus*. 

✓ Pyochelin

Pyochelin is an unusual siderophore in that it has a lower molecular mass than other bacterial or fungal siderophores [51]. It is hydrophobic and unlike pyoverdine or ferrichrome, it does not contain catecholate or hydroxamate as iron-chelating groups [64]. Pyochelin is a redox-active compound which has been shown to cause oxidative damage and inflammation in human immune cells and bacteria [65]. Pyochelin has been described to be one of the bacterial siderophores that has the best affinity for zinc [61], even if pyochelin does not behave as a zincophore for *P. aeruginosa* [66].

― Deprivation of iron and zinc from the medium by pyochelin results in an inhibition of *A. fumigatus* growth 

**Figure 2:**
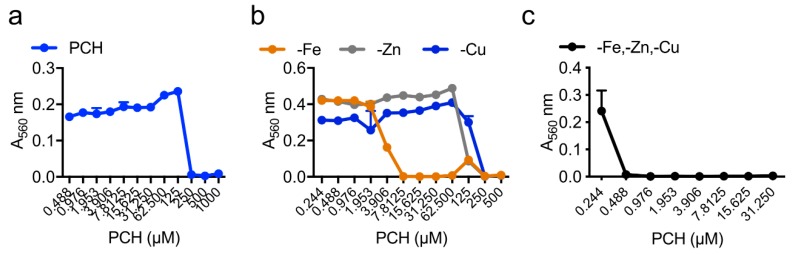
Pyochelin (PCH) antifungal activity on *A. fumigatus*. Growth was measured by the absorbance of the crystal violet-binding hyphae at 560 nm. (**a**) In minimal medium (MM); (**b**) In MM depleted in iron (-Fe), Zinc (-Zn) or copper (-Cu); (**c**) In MM depleted in iron, zinc and copper (-Fe,-Zn,-Cu). Medium composition and methodology are described in Supplementary Materials.

Pyochelin has an antifungal activity on *A. fumigatus* at 250 µM. The depletion of iron alone from the medium reduced the MIC by 35x and the joint depletion of Fe, Zn and Cu from the medium reduced the MIC by 500×, indicating that the chelation of these cations is responsible for the antifungal activity of pyochelin (Figure 2a–c). 

Pyochelin activity is pH-dependent. At pH 4.5, the pyochelin minimal inhibitory concentration (MIC) on *A. fumigatus* increases four times compared to that at pH 6.5 (MIC_pH 6.5_ = 250 µM; MIC_pH 4.5_ = 62 µM). This is probably due to the presence of the carboxyl group on pyochelin. 

Table 1 shows the effect of pyochelin on *A. fumigatus* mutants deleted in genes coding for siderophores SidA, SidC, SidD and SidF or the iron homeostasis transcription factor HapX. Siderophore pathways minus mutants Δ*sidC*, Δ*sidD* and Δ*sidF* have been used to discriminate the role of *A. fumigatus* extracellular and intracellular siderophores against pyochelin on iron or zinc-uptake. We showed that fungal mutants lacking the extracellular siderophore TAFC produced by SidD and SidF enzymes (Δ*sidD* and Δ*sidF*, respectively) were more susceptible to pyochelin for both iron and zinc pathway, while the intracellular siderophore (SidC) was not required (Table 1). In line, the strain lacking HapX, regulating both *A. fumigatus* intra- and extracellular siderophores, was also essential for iron uptake, but interestingly not for zinc uptake in the presence of pyochelin.

The triple zinc transporter mutant Δ*zrfA*Δ*zrfB*Δ*zrfC* also presented higher pyochelin sensitivity (MIC of 62 µM in MM medium) than its parental strain (MIC of 250 µM). This result confirmed the role of pyochelin in zinc uptake and depletion from the medium, inhibiting *A. fumigatus* growth (Figure 2b).

― In absence of iron, concentrations of pyochelin higher than MIC stimulate *A. fumigatus* growth

**Figure 3:**
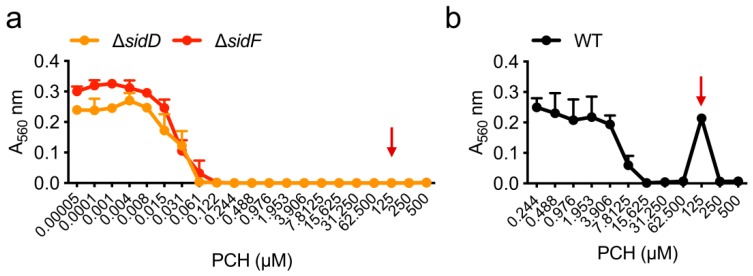
*A. fumigatus* growth stimulation (arrow) by pyochelin (PCH) sub-inhibitory concentrations. Effect of pyochelin on Δ*sidD* and Δ*sidF* in MM (**a**) and on WT in MM(-Fe) (**b**). Note the absence of pyochelin stimulation in the TAFC siderophore minus mutant’s Δ*sidD* and Δ*sidF*, showing the essentiality of the presence of TAFC. Medium composition and methodology are described in Supplementary Materials.

Surprisingly, we observed that 62 to 125 µM of pyochelin stimulate the growth of *A. fumigatus* in MM depleted in iron but not the growth of the iron mutants Δ*sidD* and Δ*sidF* in MM medium (Δ*sidD* and Δ*sidF* do not grow in MM depleted in iron) (Figure 3). This result suggested that *A. fumigatus* can use pyochelin as an external ferrochelator for iron exchange with its own siderophore TAFC (PCH pFe = 16.0; TAFC pFe = 31.8), promoting its survival when iron access is limited. Above 125 µM, pyochelin inhibited again *A. fumigatus* growth. 

― Pyochelin induces ROS production in *A. fumigatus* cells

**Figure 4:**
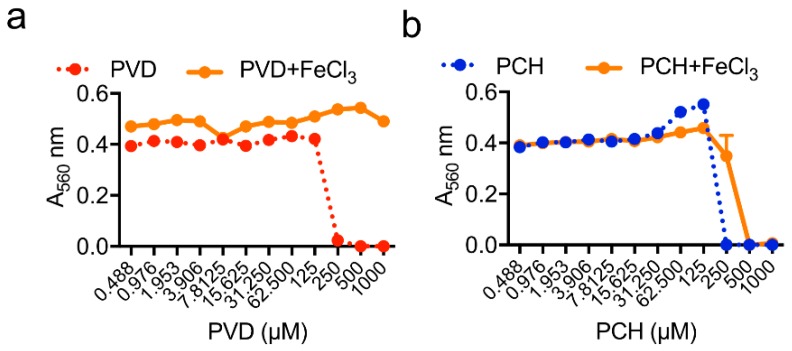
Pyoverdine (PVD) (**a**) and pyochelin (PCH) (**b**) activities on *A. fumigatus* growth in MM in presence of iron excess, showing that the antifungal effect of pyoverdine on *A. fumigatus* was abolished in presence of iron excess, whereas pyochelin antifungal activity was not abolished. Methodology is described in Supplementary Materials.

We demonstrated then that pyochelin and pyoverdine have a different antifungal mode of action on *A. fumigatus*. First, we showed that like pyochelin, pyoverdine also inhibited *A. fumigatus* growth (Figure 4a). This inhibitory effect was abolished by adding an excess of iron, showing that antifungal activity of pyoverdine is exclusively due to iron starvation. Surprisingly, an excess of iron did not abolish the antifungal effect of pyochelin on *A. fumigatus* (Figure 4b), suggesting that pyochelin, in addition to its chelating function and conversely of pyoverdine, has an additional antifungal effect. 

**Figure 5:**
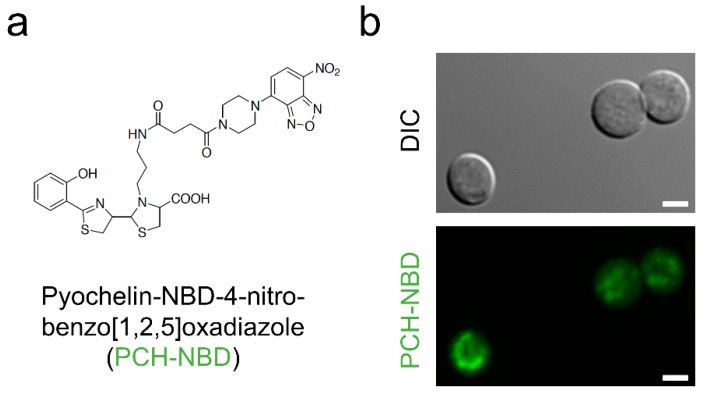
Structure (**a**) and penetration (**b**) of pyochelin-4-nitrobenzo[1,2,5]oxadiazole (PCH-NBD) in *A. fumigatus* swollen conidia. Scale bar represents 5 mm. Methodology is described in Supplementary Materials.

This result also suggested that this antifungal activity of pyochelin followed the penetration of the fungal cell. Indeed, to confirm our hypothesis, a pyochelin conjugated to the fluorochrome 4-nitrobenzo [1,2,5]oxadiazole (PCH-NBD) [67] (Figure 5a) penetrated into fungal cell as soon as the conidia underwent swelling, as shown previously for phenazines [49] (Figure 5b).

**Figure 6:**
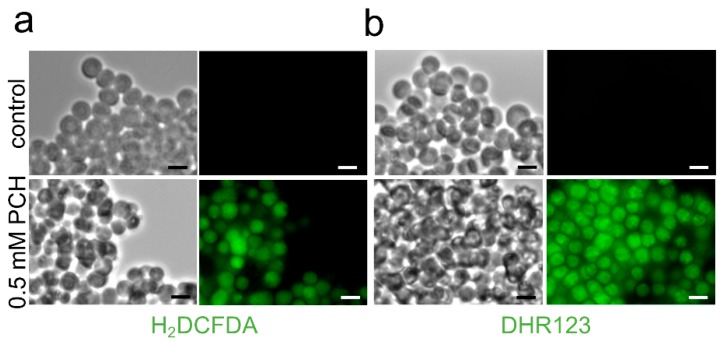
Pyochelin (PCH) induces the production of reactive oxidant (ROS) (**a**) and reactive nitrogen (RNS) (**b**) in swollen conidia using 2′,7′-dichlorodihydrofluorescein diacetate (H_2_DCFDA) and dihydrorhodamine 123 (DHR123), which are ROS- and RNS-specific fluorescent probes, respectively. Scale bar represents 5 mm. Methodology is described in Supplementary Materials.

Pyochelin has been shown to induce ROS in bacterial, endothelial and pulmonary cells [65,68]. We showed that indeed pyochelin induced ROS and RNS production by *A. fumigatus* in a similar way to phenazines (Figure 6). The production of ROS and RNS explained the resurgence of the pyochelin inhibitory effect at concentration higher than 120 µM as observed in MM(-Fe) (Figure 3b).

In conclusion, we demonstrated that pyochelin penetrates into *A. fumigatus* cells and has three distinct modes of action: two are antifungal, resulting from iron and zinc starvation and ROS-RNS induction, whereas the third one stimulated growth by acting as an external ferrochelatorfor the fungal cell.

✓ Pyoverdine

By the use of *P. aeruginosa* mutants and analysis of the effect of depletion of single or several molecules or pathways by gene deletion, Sass et al. demonstrated that pyoverdine is a major *P. aeruginosa* factor conferring antifungal activity [50]. The predominant mode of action of pyoverdine appears to be iron starvation, resulting in *A. fumigatus* inhibition and confirming our results described above in Figure 4. Pyoverdine increases *A. fumigatus* siderophores production three-fold, showing that TAFC, which have a similar pFe (pFe = 31.8), compete with pyoverdine for iron acquisition. However, conversely to pyochelin, pyoverdine cannot be used by *A. fumigatus* as an external ferrochelator [50], which is in agreement with our results.

Pyoverdine is the principal mediator of antifungal activity on *A. fumigatus* biofilms [50]. As CF progresses, much of the airway becomes hypoxic [69], and under this condition, *P. aeruginosa* produces less pyoverdine [50,69]. *P. aeruginosa* is also able to use hemin, a blood heme component, as a source of iron. The addition of hemin in *P. aeruginosa* culture medium abolished pyoverdine production by the bacteria [50]. Thus, hemin present in the blood in patient’s lungs might suppress in vivo pyoverdine production, which favors *A. fumigatus* establishment. By comparison, Lopez-Medina et al. [63], using a neutropenic mouse model of microbial gastrointestinal colonization and dissemination, showed that *C. albicans* inhibits the virulence of *P. aeruginosa* by inhibiting pyochelin and pyoverdine gene expression. No such experiments were undertaken with *A. fumigatus*.

#### 3.2.3. Pf4 *P. aeruginosa* Phage–*A. fumigatus* Interaction

Among the different bacteriophages produced by *P. aeruginosa*, one of them, Pf4, inhibited the metabolic activity of *A. fumigatus* biofilm [19]. First, the phage binds to *A. fumigatus* hyphae by an unknown mechanism, which could be GAG-mediated, similar to the binding of *P. aeruginosa* on *A. fumigatus* hyphae [70]. Second, Pf4 binds ferric iron, which has the effect of iron denial to *A. fumigatus* and inhibition of fungal growth, similarly to pyoverdine and pyochelin.

#### 3.2.4. Microbial Interaction is Promoted by Volatiles

*P. aeruginosa* and *A. fumigatus* can interact at distance via volatile-mediated communication [70]. The *P. aeruginosa* volatile compound dimethylsulfide (DMS) is responsible for the stimulatory effect on *A. fumigatus* growth. DMS contains sulfur, which serves as a nutrient source for *A. fumigatus*. Organic S-compounds are essential for the growth of *A. fumigatus*. Sulphur-volatile compounds have also been detected in sputum samples from CF patients. This volatile interaction can promote the growth of *A. fumigatus,* even if the *P. aeruginosa* and *A. fumigatus* infection sites are different in the CF patient airway. Volatiles can predispose to *A. fumigatus* co-colonization. Volatiles are also produced by *A. fumigatus*, but they have not been tested yet on bacteria [71].

### 3.3. Influence of Polymicrobial Biofilms on Drug Sensitivity

The role of mixed fungal–bacterial biofilms in the modification of the sensitivity to antifungal or antibacterial drugs has been poorly investigated. *C. albicans* and *S. aureus* are responsible for many infections in hospitalized patients and often co-isolated. It has been reported that, when grown together in a mixed biofilm, *C. albicans* provides the bacterium with enhanced tolerance to antimicrobial drugs [72]. This process was mediated by the cell wall polysaccharide β1,3 glucan secreted in the biofilm matrix, which sequestered drugs in the matrix [73].

Nothing similar has been demonstrated for mixed *A. fumigatus*–bacteria biofilms. Manuvathu et al. [30] demonstrated that mono *A. fumigatus* and mixed *A. fumigatus–P. aeruginosa* biofilms were equally susceptible to antifungal drugs such as voriconazole and posaconazole. However, *P. aeruginosa* cells are less susceptible to cefepime in *A. fumigatus–P. aeruginosa* mixed biofilm in comparison to *P. aeruginosa* biofilm [30]. The most plausible explanation is that the extracellular matrix secreted by *A. fumigatus* and embedding the bacteria [31] prevents the adequate access of cefepime to the bacteria compared to mono *P. aeruginosa* biofilm extracellular matrix [30], as observed with *C. albicans* for *S. aureus*.

### 3.4. Summary

As shown in this review, except for maltophilin, all our knowledge of *A. fumigatus*–bacterial interactions exclusively concern the role of *P. aeruginosa* molecules. Table 2 recapitulates the effect of these molecules. Negative interactions (in red in Table 2) have been observed for HSLs, pyoverdine and pyochelin resulting from iron starvation, for pyochelin and phenazine 1-HP resulting from ROS/RNS production, and for dirhamnolipids resulting in β1,3 glucan synthase inhibition. Positive interactions (in black in Table 2) have been observed for (i) dirhamnolipids which induce the formation of a thick fungal cell wall enriched in the immunomodulatory molecule GAG, in chitin and melanin, modifying the drug diffusion and *A. fumigatus* susceptibility; (ii) phenazines which at biological concentrations (<100 µM) [74] enhance fungus growth with ferrous iron uptake; (iii) pyochelin, which at sub-inhibitory concentrations can be used by *A. fumigatus* as an external ferrochelator; and (iv) the volatile DMS secreted by *P. aeruginosa*, which is an essential component for *A. fumigatus* growth. On the host cells, all *P. aeruginosa* molecules have negative impact (Table 2).

## 4. How are Mixed Bacterial–Fungal Infections Seen by the Host Immune Response?

### 4.1. Fungal and Bacterial Metabolites Influence the Host Immune Response

However, the host immune response to polymicrobial infections remains insufficiently investigated. Whether the dual infection stimulates both pathogen-specific immune pathway responses or is dominated by one pathogen-specific response has only just started to be explored. Developing an understanding of how the host immune system responds to polymicrobial infection may help elucidate disease mechanisms and uncover new insights for novel therapeutic strategies. Allard et al. [75] reported that oropharyngeal instillation of *C. albicans* or *A. fumigatus* lysates without previous immunization and in the absence of adjuvant led to airway eosinophilia, release of Th2-type cytokines and changes in mucus production as in allergic bronchopulmonary aspergillosis (ABPA). In contrast, bacterial antigens from *P. aeruginosa* induced an inflammatory response dominated by neutrophils recruitment and secretion of Th1 type cytokines with minimal mucus production [75]. Many molecules produced by *P. aeruginosa* can be responsible in vivo for the pathophysiological responses such as HSLs in inflammation [52], host immune cells apoptosis due to ROS production induced by phenazines, pyochelin and rhamnolipids [37,58,65,68,74,76]. Co-administration of same amount of bacterial and fungal antigens activated immune responses typical of the single bacterial antigens [75]. However, these results only concern immune responses to a certain amount of molecules, which can be very different to the concentration found in vivo during an infection, and we know now that microorganisms interact to establish equilibrium between them and can modulate the growth of the other partner and the production of new molecules.

### 4.2. In Vivo/Ex Vivo Models

Co-infections and persistence of the two microorganisms in a host is difficult to attain in in vivo models because of the fast killing of the animal by the dissemination of one partner. Nevertheless, the development of such models is necessary to decipher the immunological perturbations resulting from the cohabitation of the bacterial and fungal partners. Two chronic pulmonary mouse and one *Galleria mellonella* models of co-infection by *M. abcessus* or *P. aeruginosa* and *A. fumigatus* have been described [15,24,77]. However, in the first *M. abcessus*–*A. fumigatus* mouse model, the mice were not immunosuppressed and there was no infection by *A. fumigatus* [15]. In the second model of *P. aeruginosa–A. fumigatus* coinfection, mice were immunosuppressed and *A. fumigatus* was embedded in agar beads, which had the advantage of allowing 16 days of chronic development of the fungus before invasive aspergillosis and killing of the mice [24]. Rat or murine models of chronic *P. aeruginosa* pneumonia, using agar beads containing *P. aeruginosa* have been described but they do not include a co-infection with *A. fumigatus* [78,79]. However, all these models do not mimic co-infections in COPD or CF patients where the genetic and pathophysiological mechanisms are key drivers of fungal and bacterial persistence. The only CF model described is *A. fumigatus* inhalation in CFTR^−^/^−^ mice [80]. However, mice with deletion in the CFTR gene do not recapitulate human CF disease [81]. CFTR^−^/^−^ pigs or ferrets developed lung disease characterized by airway inflammation, mucus accumulation and infection with multiple bacterial species as in CF patients, but *P. aeruginosa* was absent in the lungs of these animals and fungal infections were not reported, making this model unsuitable for studying *A. fumigatus–P. aeruginosa* interactions [81]. Consequently, a good model of chronic co-infections by *A. fumigatus* and bacteria does not exist to date.

Ex vivo/in vitro host immune cellular models in which host cells are infected in multi-well plates by bacteria and *A. fumigatus* have been developed. These models use immortalized lung cell lines (in vitro immune cell models) or monocytes or differentiated host immune cells purified after removal from the host (*ex vivo* immune cell models). However, *A. fumigatus* or the bacteria growth in these systems cannot be controlled and induces cell death after a short period of time. They therefore represent more of an infection process than a sustained colonization. Most ex vivo studies have been done with one live microorganism and antigens of the partner, or killed microorganisms. For examples, in one ex vivo model of *M. abscessus* infecting macrophages, fungal products were added prior the bacterial infection and activation of macrophages was investigated [15], and in the other ex vivo model, killed *P. aeruginosa* and *A. fumigatus* antigens were used to stimulate CF peripheral blood mononuclear cells (PBMC), and cytokines were quantified [82]. Only one in vitro immune cell model of co-infection with different strains of alive *P. aeruginosa* and *A. fumigatus* in CF bronchial epithelial cell lines (CFBE cells derived from a cystic fibrosis patient homozygous for the ΔF508 CFTR mutation) has been reported [77]. After 24 h co-infection, supernatants were removed and used for pro-inflammatory assays. In our laboratory, we developed a model in which live *P. aeruginosa* first infected macrophages, followed by the inoculation of live conidia of *A. fumigatus* (see Supplementary Materials for a precise description of the experimental method). After 16 h of co-infection, the cytokine production was quantified (Rasoldier, Briard, Hatinguais, Quintin and Beauvais, unpublished results). We add our data in this review because it is the first ex vivo model in which we used live microorganisms.

### 4.3. Immune Response in Animal Model of Co-Infection

In mono-infection, *A. fumigatus* induced IL17 cytokines and neutrophilia which has been associated with inflammation and impaired immune response. IL17 is beneficial for *A. fumigatus* persistence, given that it inhibited Th1 responses required to control infection and promote fungal biofilm formation [15]. In mono-infection, *M. abscessus* dysregulated the immune response and the presence of dead or dying neutrophils enhanced the capacity of *M. abscessus* to form a biofilm [83]. Mice co-infected with *M. abcessus* and *A. fumigatus* exhibited lung inflammation but improved clearance of *M. abcessus*. This improved control was partly dependent on IL17RA and STAT1 signaling in addition to high expression of T-bet and RORγt, which are transcription factors for the Th1 and Th17 responses, respectively. In the second model, agar beads containing *A. fumigatus* conidia were inoculated prior to immunosuppression and *P. aeruginosa* infection [24]. This model allows the histological observation of the two species and host response in lungs. A higher proliferation of *A. fumigatus* hyphae from the agar beads in the lungs of co-infected mice was observed compared to *A. fumigatus* mono-infected mice, and the bacterial CFU in the lungs of co-infected mice was higher than in *P. aeruginosa* mono-infected mice. Thickening of the interalveolar septum, bleeding and infiltration of inflammatory cells were observed but without significant difference between mono- or co-infections [24].

The pathogenesis of *A. fumigatus* and *P. aeruginosa* during a co-infection was also evaluated in the *Galleria mellonella* acute infection model [77]. *G. mellonella* were inoculated with non-lethal doses of *A. fumigatus* strains 24 h prior to subsequent inoculation with *P. aeruginosa* strains. Pre-exposure of larvae to *A. fumigatus* resulted in an increase in virulence of *P. aeruginosa*.

In CF patients who are unable to easily clear infection due to CFTR-deficient phagocytes against microorganisms, the increased lung inflammation and damage will contribute to a decline in pulmonary function. In CFTR^−^/^−^ mice, the fungus promoted exaggerated lymphocytic inflammation, mucin accumulation, and lung injury [80]. However, co-infection with *P. aeruginosa* has never been performed in this model.

### 4.4. Immune Response in In Vitro and Ex Vivo Models of Co-Infection

In the model of CF epithelial cells, the joint inoculation of one *P. aeruginosa* clinical isolate and one *A. fumigatus* clinical isolate resulted in a significant increased production of pro-inflammatory cytokines IL6 and IL8 [77]. The lack of enhancement of pro-inflammatory responses for the majority of tested co-infections with other *P. aeruginosa* and *A. fumigatus* clinical strains suggests that their association may not generally further exacerbate the inflammatory response, compared to mono-cell infection [77]. In the ex vivo model of *A. fumigatus* and *P. aeruginosa* stimulating PBMC, secretion of increased amount of the anti-inflammatory IL10, inhibiting *A. fumigatus* T cell responses, was observed, suggesting an adaptive mechanism observed in CF patients in response to prolonged cycles of lung inflammation and damages due to infections [82]. Using the ex vivo model of co-infected by *M. abscessus* and fungal antigens, the authors demonstrated that β1,3 glucans treatment of macrophages improved *M. abscessus* control [15].

**Figure 7:**
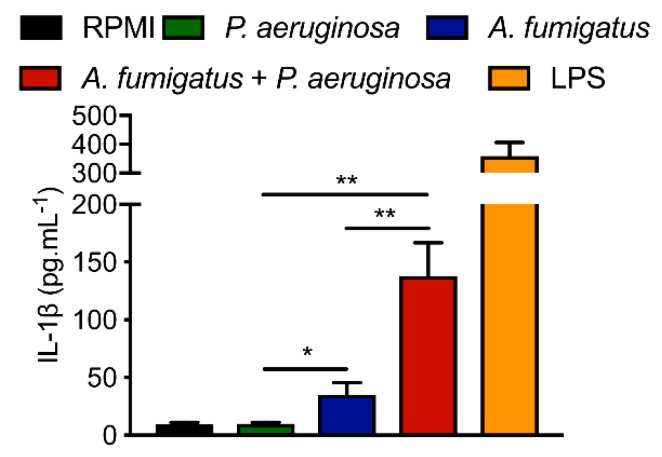
Previous bacterial challenge synergistically increases the production of IL1β in response to *A. fumigatus*. Monocytes were stimulated by RPMI medium (in black), bacterial lipopolysaccharide (LPS, 10ng/mL, in orange), *P. aeruginosa* alone (in green), *A. fumigatus* alone (in blue), *P. aeruginosa–A. fumigatus* sequential infection (in red) according to protocol described in Supplementary Materials. Stars represent statistical difference (* *p* < 0.05; ** *p* < 0.01).

In our ex vivo model with macrophages, we observed that sequential infection of monocytes by *P. aeruginosa* and *A. fumigatus* synergistically increased the secretion of the pro-inflammatory cytokine IL1β (Figure 7). Under the same experimental conditions, the production of TNFα by macrophages was additively but not synergistically increased after co infection. IL6 secretion was similar in *P. aeruginosa*–*A. fumigatus* sequential infection compared to mono *P. aeruginosa* or *A. fumigatus* infections. The synergistic increase in IL1β release during co-infection could explain the dramatic decrease in respiratory functions of CF patients colonized by both pathogens due to an over-inflammatory environment [23].

## 5. Perspectives

As shown in this review, most data on *A. fumigatus* and bacterial lungs was obtained with *P. aeruginosa* during in vitro confrontations. Studies on other bacteria and the consideration of the host responses in CF and other diseases are only starting to be analyzed. We showed that *P. aeruginosa* has antifungal or fungal growth stimulating effects on *A. fumigatus*. In vivo, a balance between these two opposite actions should also exist in CF patients co-infected by *A. fumigatus* and bacteria, as it was observed that intravenous antibiotics targeting *P. aeruginosa* during CF pulmonary exacerbations reduces the presence of *A. fumigatus* in the patients [84]. In contrast, Burns et al. [85] observed that exposure to frequent antibacterial therapy increases pulmonary fungal load. In addition, in pulmonary diseases, much of the airway is hypoxic or anaerobic [69]. In the areas of hypoxia, the ratio Fe^3+^ to Fe^2+^ decreases, inducing a change in *P. aeruginosa* metabolism with for example a decrease in pyoverdine production which is replaced by phenazines for Fe^2+^ transport into the bacterial cells [51]. The bacterial metabolites are also less inhibitory towards *A. fumigatus* growth and biofilm formation than those under aerobic conditions, promoting fungal growth [69]. Moreover, changes in the environmental conditions will lead to the selection of mucoid or non-mucoid isolates of *P. aeruginosa*, which have a different inhibitory capacity, especially against *A. fumigatus* biofilm [25]. Therefore, there is an urgent need to develop in vivo animal models for pulmonary co-infection with immunological disorders such as CF, COPD, ABPA and chronic granulomatous disease, with bacterial or viral preexisting infections. Since in vivo models of infection mimicking human diseases with all their associated pathophysiological adjustments will be difficult to implement, ex vivo models in trans-well inserts is feasible and would have the advantage of growing hyphae and bacteria in the presence of host cells. Organoid models recently developed could be an interesting alternative [86,87]. These models will present also the advantage of analyzing *A. fumigatus* molecules specifically produced in contact with the host and the bacteria. 

The impact of the different bacterial lung inhabitants on the production of *Aspergillus* secondary metabolites has been ignored, thus far, whereas it has been shown that soil bacterial species such as *Streptomyces peucetius*, *S. bullii* or *S. rapamycinicus* modify the secretion of *Aspergillus* secondary metabolites. These bacteria led *Aspergillus* to produce formyl xanthocillin, ergosterol or to upregulate meroterpenoïd pathway [88,89,90]. The activation of new secondary metabolite pathways that are normally silent was mediated by manipulating the chromatin-based regulation in *A. fumigatus* by the bacteria [88]. Such modifications of the secondary metabolite spectrum during host infection in contact with bacteria could contribute to modifications of the host immune defense. In addition, no studies have investigated the role of secreted *A. fumigatus* molecules (mycotoxin but also enzymes) on bacterial metabolism.

More and more data show that the gut microbiota has a profound impact on the lung microbiota [91]. Alterations of the gut microbiome by antibiotics predispose to *A. fumigatus*-induced allergic airway disease in murine model [9]. The gut microbiome and its metabolites can modulate pulmonary host defense: immune pulmonary responses induced by *A. fumigatus* infection are influenced by the composition of the microbiome [91]. Accordingly, the investigation of the links between lung and gut microbiota in specific *Aspergillus* lung diseases such as CF, COPD, ABPA and invasive aspergillosis should open up new research avenues in Aspergillosis. If microbial ecology has recently become a major concern in human health, it is time to take better account of its role in in medical mycology and especially for a better understanding of the different forms of aspergillosis in humans.

## Figures and Tables

**Table 1:** Pyochelin minimal inhibitory concentration (MIC) on WT and mutants deleted in iron-stress responses (Δ*hapX*) or siderophore (Δ*sidC,* Δ*sidD,* Δ*sidF*) in MM medium or MM depleted in iron MM(-Fe), zinc MM(-Zn). Medium composition and methodology are described in the Supplementary Materials.

Strain	MM	MM(-Fe)	MM(-Zn)
WT	250 µM	7.8 µM	250 µM
Δ*hapX*	250 µM	1.9 µM	250 µM
Δ*sidC*	250 µM	7.8 µM	250 µM
Δ*sidD*	62 µM	0.1 µM	0.8 µM
Δ*sidF*	62 µM	0.1 µM	0.8 µM

**Table 2:** Bacteria–*A. fumigatus*–host interactions. Effect of *P. aeruginosa* molecules on *A. fumigatus* and host immune cells. In grey, antifungal and anti-host cell activity by *P. aeruginosa* molecules; in blue, stimulation of *A. fumigatus* growth by *P. aeruginosa* molecules.

*P. aeruginosa*	*A. fumigatus*	Host Immune Cells
Homoserine lactones	Fungal growth inhibition	Interferon-γ induction, NFκB disruption
Pyoverdine	Iron starvation, fungal growth inhibition	?
Pyochelin	Iron starvation, ROS/RNS production, fungal killing	ROS production, cell apoptosis
	Ferric iron provision, fungal growth stimulation	
Phenazine 1HP	Iron chelation, ROS/RNS production, fungal killing	ROS production, cell apoptosis
Phenazines PYO, PCA, PCN (< 100 µM)	Ferric iron provision, fungal growth stimulation	ROS production, cell apoptosis
Dirhamnolipids	Inhibition of β1,3 glucan synthase	Polymorphonuclear leucocytes necrosis, calcium-mediated protein kinase C inhibition
	Thick cell wall, high chitin, GAG and melanin production, persistence, resistance to caspofungin	
Dimethylsulfide	Fungal growth stimulation	?

1HP, 1-hydroxy-phenazine; PYO, pyocyanin; PCA, phenazines-carboxylic acid; PCN, phenazines-carboxamide; ROS, reactive oxidant species; RNS, reactive nitrogen species; GAG, galactosaminogalactan.

## Supplementary Materials

The following are available online at https://www.mdpi.com/2309-608X/5/2/48/s1, File S1: Effect of *P. aeruginosa* siderophore pyochelin and pyoverdine on *A. fumigatus*, File S2: *Ex vivo* human macrophage model of co-infection with *P. aeruginosa* and *A. fumigatus*.
