# Supplementary material for: Interactions between Aspergillus fumigatus and Pulmonary Bacteria: Current State of the Field, New Data, and Future Perspective
Source: J Fungi (Basel). 2019 Jun 12;5(2):48. doi: 10.3390/jof5020048 (PMC6617096; doi:10.3390/jof5020048)
Supplement: Supplementary file 1 [file jof-05-00048-s001.pdf]

Journal of
Fungi

Supplementary informations

1. Effect of *P. aeruginosa* siderophore pyochelin and pyoverdine on *A. fumigatus*.

a. Strains used in this study

**Souches**	**Génotype**	**Reference**
Parental strain: CEA17Δ*akuB*KU80	CEA17Δ*akuB*KU80	[1]
Parental strain: ATCC 46645	Souche sauvage	American Type Culture Collection
Δ*hapX*	ATCC 46645ΔhapX::hph	[2]
Δ*sidC*	ATCC 46645ΔsidC::hph	[3]
Δ*sidD*	ATCC 46645ΔsidD::hph	[3]
Δ*sidF*	ATCC 46645ΔsidF::hph	[3]
Parental strain: AF14	Souche sauvage	[4]
Δ*zrfA*Δ*zrfB*Δ*zrfC*	AF14	[5]

b. Medium composition

The medium used to test the effect of pyochelin on *A. fumigatus* growth was minimal medium (MM) containing 10g/L glucose, 0.92g/L ammonium tartrate dibasic, 1ml/L trace-element-solution and 20ml/L 50x salt solution, pH 6.5. Trace-element-solution contained Na2B4O7 x10 H2O 40 mg/L, CuSO4 x5 H20 400 mg/L, MnSO4 x4 H20 800 mg/L, Na2MoO4 x10 H20 800 mg/L, ZnSO4 x7 H2O 8g/L, 5 µM $FeCl_3$ (2 M stock solution in 1 M HCl) and 0.2 mM HCl. Salt solution contained KCl 26 g/L, MgSO4 x7 H20 26 g/L and KH2P04 76 g/L). To get an excess of iron in MM, 250 µM $FeCl_3$ were added.

c. Minimal Inhibitory Concentration (MIC) determination

The susceptibility of *A. fumigatus* to pyochelin or pyverdine was undertaken by measuring the MIC in 96-well flat bottom plates. Briefly, the assay mixture was prepared by adding 1 volume of conidial suspension ($1x10^5$ conidia/mL in 0.05% Tween20-water) to 12 volumes of assay medium (MM containing 0.1% Tween 20, and 1% methanol. Twofold dilutions of pyochelin or pyoverdine were prepared with this assay mixture. The plates were incubated at 37°C for 20 h. Biofilm biomass was assessed using crystal violet protocol previously described in [6]. Briefly, after incubation, the culture medium was removed and the biofilms were washed with water. 130 ml of 0.01% (w/v) crystal violet solution (Sigma Aldrich) was added to each well for 20 min at room temperature. The solution was then removed and the biofilms were washed with water until the supernatant was clear. The plates were air-dried. The biofilms were destained by the addition of 130 ml of 30% acetic acid in for 20 min under agitation. The acetic acid was transferred to clean 96 well plates and the absorbance at 560 nm was measured (Thermo Labsystems Multiskan EX).

d. Penetration of pyochelin in *A. fumigatus* cell

To test the penetration of pyochelin-4-nitrobenzo[1,2,5]oxadiazole (PCH-NBD) in *A. fumigatus*, swollen conidia were obtained after 4 h 30 min incubation into MM at 37°C under shaking and then incubated with 250 µM PCH-NBD (pyochelin MIC) in MM for 1 h 30 min at 37°C. the fluorescence was observed by Epifluorescence microscopy using the filter excitation 472 ± 30 nm, emission 520 ± 30 nm and dichroic 502-730 nm.

e. Reactive oxidants species (ROS) and reactive nitrogen species (RNS) assays

To test the induction of ROS and RNS in *A. fumigatus* cells by pyochelin, we used the $O_2^{\cdot -}$ fluorescent probe using 2',7'-dichlorodihydrofluorescein diacetate (H2DCFDA) and dihydrorhodamine 123 (DHR123) probes which become fluorescent in presence of ROS and RNS respectively following the method described previously for phenazines inducing ROS and RNS in *A. fumigatus* [6]. Briefly, H2DCFDA and DHR123, ROS and RNS fluorescent probes respectively, were added to swollen conidia followed by the addition of 250 µM pyochelin for 1 h in the darkness at 37°C. Fluorescence of the respective reduced products DCF and Fluorescein 123 was visualized using a fluorescence microscope Leica DMLB with Leica filter I3, a filter excitation BP 450-490 nm, FT 510 nm and emission LP 515 nm. For control, swollen conidia were incubated with H2DCFDA and DHR123 in absence of pyochelin.

2. *Ex vivo* human macrophage model of co-infection with *P. aeruginosa* and *A. fumigatus*

In multi-well plates , human GM-CSF or M-CSF differentiated monocyte-derived macrophages [7] were growing in RPMI 1640 supplemented with 10% fetal bovine serum (Sigma) until confluence and then infected with *P. aeruginosa* (2.5x105 CFU/mL or MOI 0.05) for 2 h. Bacteria were eliminated by renewing the medium and adding tobramycin, an aminoglycoside antibiotic used in clinic to treat *P. aeruginosa* infection. After 90 min, supernatants were removed and macrophages infected with *A. fumigatus* (2.5x106 conidia/mL or MOI 10). Cells were incubated overnight and supernatant collected and used for cytokines quantification [7].

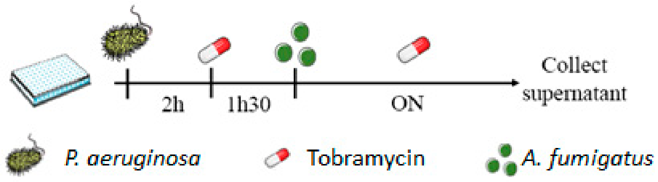

References

1. Ferreira, M.E. da S.; Kress, M.R.V.Z.; Savoldi, M.; Goldman, M.H.S.; Härtl, A.; Heinekamp, T.; Brakhage, A.A.; Goldman, G.H. The akuBKU80 Mutant Deficient for Nonhomologous End Joining Is a Powerful Tool for Analyzing Pathogenicity in Aspergillus fumigatus. *Eukaryot. Cell* **2006**, *5*, 207–211.

2. Schrettl, M.; Beckmann, N.; Varga, J.; Heinekamp, T.; Jacobsen, I.D.; Jöchl, C.; Moussa, T.A.; Wang, S.; Gsaller, F.; Blatzer, M.; et al. HapX-mediated adaption to iron starvation is crucial for virulence of Aspergillus fumigatus. *PLoS Pathog.* **2010**, *6*, e1001124.

3. Schrettl, M.; Bignell, E.; Kragl, C.; Sabiha, Y.; Loss, O.; Eisendle, M.; Wallner, A.; Arst, H.N.J.; Haynes, K.; Haas, H. Distinct roles for intra- and extracellular siderophores during Aspergillus fumigatus infection. *PLoS Pathog.* **2007**, *3*, 1195–1207.

4. Vicentefranqueira, R.; Moreno, M.A.; Leal, F.; Calera, J.A. The zrfA and zrfB genes of Aspergillus fumigatus encode the zinc transporter proteins of a zinc uptake system induced in an acid, zinc-depleted environment. *Eukaryot. Cell* **2005**, *4*, 837–848.

5. Amich, J.; Vicentefranqueira, R.; Mellado, E.; Ruiz-Carmuega, A.; Leal, F.; Calera, J.A. The ZrfC alkaline zinc transporter is required for Aspergillus fumigatus virulence and its growth in the presence of the Zn/Mn-chelating protein calprotectin. *Cell. Microbiol.* **2014**, *16*, 548–564.

6. Briard, B.; Bomme, P.; Lechner, B.E.; Mislin, G.L.A.; Lair, V.; Prévost, M.-C.; Latgé, J.-P.; Haas, H.; Beauvais, A. Pseudomonas aeruginosa manipulates redox and iron homeostasis of its microbiota partner Aspergillus fumigatus via phenazines. *Sci. Rep.* **2015**, *5*, 8220.

7. Camilli, G.; Eren, E.; Williams, D.L.; Aimanianda, V.; Meunier, E.; Quintin, J. Impaired phagocytosis directs human monocyte activation in response to fungal derived β-glucan particles. *Eur. J. Immunol.* **2018**, *48*, 757–770.
